# Gender-specific associations between perceived and objective neighbourhood crime and metabolic syndrome

**DOI:** 10.1371/journal.pone.0201336

**Published:** 2018-07-26

**Authors:** Katherine L. Baldock, Catherine Paquet, Natasha J. Howard, Neil T. Coffee, Anne W. Taylor, Mark Daniel

**Affiliations:** 1 Centre for Population Health Research, School of Health Sciences, Sansom Institute for Health Research, University of South Australia, Adelaide, South Australia, Australia; 2 Wardliparingga Aboriginal Research Unit, Sansom Institute for Health Research, University of South Australia, Adelaide, South Australia, Australia; 3 Centre for Research & Action in Public Health, Health Research Institute, Faculty of Health, University of Canberra, Canberra, Australian Capital Territory, Australia; 4 Population Research and Outcome Studies, Discipline of Medicine, The University of Adelaide, Adelaide, South Australia, Australia; 5 Department of Medicine, St Vincent’s Hospital, The University of Melbourne, Melbourne, Victoria, Australia; 6 South Australian Health and Medical Research Institute, Adelaide, South Australia, Australia; University of Bristol, UNITED KINGDOM

## Abstract

Much research has considered the relationship between neighbourhood crime and physical activity, but few studies have assessed clinical outcomes consequent to behaviour, such as cardiometabolic risk. Fewer still have simultaneously assessed perceived and objective measures of crime. Perceptions of crime and actual victimisation vary according to gender; thus, this study sought to assess: 1) correspondence between perceived and objective neighbourhood crime; and 2) gender-specific associations between perceived and reported crime and metabolic syndrome, representing cardiometabolic risk. The indirect effect of neighbourhood crime on metabolic syndrome via walking was additionally evaluated. An Australian population-based biomedical cohort study (2004–2007) collected biomedical, socio-demographic, and neighbourhood perceptions data from n = 1,172 urban-dwelling, adults. Area-level reported crime rates were standardised and linked to individual data based on participants' residential location. Correspondence between actual and perceived crime measures was assessed using Pearson correlation coefficients. Cross-sectional associations between crime and metabolic syndrome were analysed using generalised estimating equations regression models accounting for socio-demographic factors and area-level income. Correspondence between perceived and objective crime was small to medium among men and women (*r* = 0.17 to 0.33). Among men, metabolic syndrome was related to rates of violent (OR = 1.21, 95% CI 1.08–1.35) and total crime (OR = 1.17, 95% CI 1.04–1.32), after accounting for perceived crime. Among women, metabolic syndrome was related to perceived crime (OR = 1.35, 95% CI 1.14–1.60) after accounting for total reported crime. Among women, there were indirect effects of perceived crime and property crime on metabolic syndrome through walking. Results indicate that crime, an adverse social exposure, is linked to clinical health status. Crime rates, and perceptions of crime and safety, differentially impact upon cardiometabolic health according to gender. Social policy and public health strategies targeting crime reduction, as well as strategies to increase perceptions of safety, have potential to contribute to improved cardiometabolic outcomes.

## Introduction

Neighbourhood crime and perceived safety are important indicators of the residential social environment. Much research has considered the relationship between neighbourhood crime and physical activity, primarily walking behaviour [1–10]; however, fewer studies have considered outcomes beyond behaviour, such as cardiometabolic risk conditions like metabolic syndrome, a clustering of factors (including for instance obesity, high blood pressure and dyslipidaemia) predictive of cardiovascular and metabolic diseases [11–13]. Two studies using objectively measured, actual area crime rates found evidence of links to cardiovascular risk [14] and coronary heart disease [15]. Perceived neighbourhood crime and safety has been found by some studies to relate to body mass index (BMI) [16, 17], obesity [18], metabolic syndrome [19], and an index indicating the sum of self-reported chronic health conditions (such as respiratory problems, cancer, cardiovascular conditions, sleep problems, and depression and anxiety) [20]. Not all results from studies of perceived crime have been consistent–null associations with BMI [21] and acute myocardial infarction [22] have also been reported. Variations in the measures used to represent neighbourhood crime and safety, i.e., objective versus perceived measures, challenge any overall generalisation about the relationships between crime and health status.

In health-related research, crime and safety are typically expressed as reported crime rates, local-area physical disorder (for example, litter, graffiti and abandoned buildings), or resident perceptions of local-area crime, safety and disorder, or fear of crime. Perceptual measures are often used to represent area features in health research, particularly where objective data are difficult to obtain [23]. Objective measures of crime such as crime rates reflect the actual level of crime within a given area, usually administratively defined. Perceived crime assessed for individuals, however, likely reflects an individual’s cognitive evaluation of both the level of crime *and* exposure to crime within the perceived boundaries of one’s local area [24, 25]. It is also shaped by emotions such as fear and mistrust that accompany negative perceptions of crime and feelings of safety [26].

Evidence to date indicates that a correspondence between objective and perceived measures of environmental attributes is weak [8, 27], suggesting differences in the underlying constructs being measured [22]. As such, inclusion of both perceived and objective measures of environmental features in place-health research is recommended [10, 28], and important, given the implications for environmental interventions aimed at improving population health. For example, strategies aimed at reducing actual crime rates may not be effective in improving cardiometabolic health at the population level if perceived crime rates are causally related to population health, and residents continue to perceive high levels of crime or a lack of safety in their local area. No study to our knowledge has evaluated associations between perceived *and* objective measures of neighbourhood crime and any cardiometabolic outcome beyond obesity in a population-based sample.

The notion that crime is related to cardiometabolic outcomes is not implausible. Daily exposure to a stressful local environment characterised by crime could impact cardiometabolic health through perpetuated physiological stress responses and their maladaptive impacts [29, 30]. Neighbourhood crime and safety may also lead to poor cardiometabolic health through a cognitive processing of perceived threatening environmental stimuli that may result in reduced levels of physical activity [29, 31, 32], a risk factor for metabolic syndrome [33, 34] and other clustered metabolic risk outcomes [35]. A previous study showed that physical activity partially mediated an association between perceived safety from crime and obesity [36]; thus, physical activity may mediate an association between crime and cardiometabolic risk.

Crime and cardiometabolic health associations, and mediating pathways, may differ according to gender. It is well-established that women experience a greater fear of crime, and perceive a greater risk of crime and victimisation than men [26, 37–40], and feeling unsafe in one’s neighbourhood has been associated with lesser physical activity for women but not men [5, 6]. Perceptions of crime and insecurity among women could therefore lead to cardiometabolic outcomes through either reduced physical activity [31], or a physiological stress response [29]. Men, on the other hand, are more likely to experience victimisation from crime compared to women [39], and neighbourhood violence has been associated with lower levels of physical activity for men but not women [5]. High crime rates have also been related to greater perceived stress among men but not women [41]. Similarly, greater exposure to actual crime has been associated with a greater odds of reporting fair-to-very-bad self-rated health for men, but not women [42]. It is possible, therefore, that objectively measured crime rates may be related to cardiometabolic outcomes for men, while perceptions of crime may be related to cardiometabolic outcomes for women.

This study used a population-based urban sample of Australian adults to examine relationships between perceived and objective measures of crime and metabolic syndrome, a measure of cardiometabolic risk. The primary aims were to assess: (1) the correspondence between perceived and objective crime measures; and (2) the associations between crime and metabolic syndrome, according to gender. To strengthen the evidence for a potential causal effect of crime on metabolic syndrome [43] a secondary aim was to test for an indirect effect of objective and perceived crime on metabolic syndrome via walking behaviour.

### Methods

#### Study context

Cross-sectional associations between crime and metabolic syndrome were evaluated as part of the Place and Metabolic Syndrome (PAMS) project. The PAMS project used data previously collected from participants in the second wave of a biomedical cohort study, the North West Adelaide Health Study (2004–2007) conducted in Adelaide, South Australia, and crime statistics routinely collected by the South Australian Office of Crime Statistics and Research [44]. The PAMS project was approved by the Ethics of Human Research Committees of the Central Northern Adelaide Health Service, the University of South Australia, and the South Australian Department of Health and Ageing.

The Adelaide metropolitan area stretches 80 km north-south and 30 km east-west of the city centre. It has a population of around 1.1 million persons, equating to 72% of the total South Australian population [45]. In Adelaide, in 2006, approximately 15% of the population were aged 65 years or older, 48% were married, 24% had been born outside Australia, 89% were employed, and the median weekly household income was AUD 924 [46]. Adelaide's total reported crime rate in 2006 was 208.3 per 1,000 persons [44]. For the same year, its violent crime (homicides, assaults, and sexual assaults) rate was 14.6 per 1,000 persons, and its property crime (burglaries, break and entries, serious criminal trespassing, larceny, arson, and property damage) rate was 123.7 per 1,000 persons.

#### Sample

Individual-level socio-demographic characteristics, clinically measured components of metabolic syndrome, and perceptions of local-area crime were obtained from participants in the North West Adelaide Health Study (NWAHS). The NWAHS is a longitudinal cohort of randomly selected adults aged 18 years and over (n = 4,056), originally recruited between 2000 and 2003 (Wave 1) from the northern and western metropolitan regions of Adelaide. Detailed methods for the NWAHS, including recruitment and response rates and data collection, have been reported elsewhere [47, 48]. NWAHS data collected across Wave 2 (2004–2007; n = 3,205) were utilised for this cross-sectional analysis, as this was the only interval over which all required measures were available.

Approximately 94% of NWAHS participants who had moved residence between Waves 1 and 2 were still residing in the north-west region of Adelaide at Wave 2. Participants provided self-reported socio-demographic information via telephone interview and written questionnaire, and attended a clinic where biomedical measurements were taken. Information on current medications prescribed for participants was obtained by linking Australian Pharmaceutical Benefits Scheme data to each participant using their Medicare number. In a follow-up of the main NWAHS cohort after the Wave 2 clinic visit, n = 1,943 participants completed an additional questionnaire which elicited their perceptions of local-area features including crime and safety.

### Measures

#### Outcome variable

Metabolic syndrome is a clustering of cardiometabolic risk factors predictive of developing type 2 diabetes and cardiovascular disease [11–13], and is useful for assessing population-level risk for cardiometabolic diseases [49]. Metabolic syndrome was classified using the International Diabetes Federation (2006) criteria, including central obesity (defined as waist circumference ≥80cm for women, and ≥94cm for Europid men or ≥90cm for non-Europid men) plus any two of the following four factors: raised triglyceride level (>1.7 mmol/L); reduced HDL cholesterol (<1.03 mmol/L in men and <1.29 mmol/L in women), or treatment for lipid abnormality; raised blood pressure (systolic blood pressure ≥130 or diastolic blood pressure ≥85 mm Hg), or treatment for hypertension; raised fasting plasma glucose (FPG; ≥5.6 mmol/L), or previously diagnosed type 2 diabetes. Criteria for dyslipidaemia or hypertension were considered met if a participant had been prescribed medication to treat such conditions in the six months prior to their clinic attendance.

#### Independent variables

Independent variables included perceived neighbourhood crime and reported crime rates (including total crime, property crime, and violent crime).

Perceived neighbourhood crime was operationalised using six items from the Australian version of the Neighbourhood Environment Walkability Scale (NEWS-AU) [50], a modified version of the NEWS [51]. A factor analysis undertaken on the NEWS-AU items [19] generated six items that loaded on a crime factor (Cronbach’s alpha = 0.80). The crime factor expresses responses to perceptions of crime in the local area (defined in the NEWS-AU as being within a 10–15 minute walk from home), for example, “There is a lot of petty crime in my local area (e.g., vandalism, shoplifting)”, and “The level of crime in my local area makes it unsafe to walk during the day”. The standardised factor score was used to represent perceived crime in the local area. A higher score indicates a higher level of perceived crime.

Objective crime data for this study were obtained from the South Australian Office of Crime Statistics and Research [44] for the year 2006, aggregated to the Statistical Local Area (SLA) and expressed as rates per 1,000 persons. The SLA is a large, administratively defined spatial unit, designed for population planning purposes, and is predominantly used to disseminate statistics other than those collected from the Population Censuses [52]. In 2006, metropolitan Adelaide SLAs had a mean resident population of 20,478 (range = 3368–34861), and varied in size from 3.9 to 339.2 square kilometres (km^2^), with a mean of 33.2 km^2^ [52]. The SLA was the smallest spatial unit at which crime data were available for this study.

Three measures of reported crime rates were examined in this study: 1) violent crime, including homicides, assaults, and sexual assaults; 2) property crime, including burglaries, break and entries, serious criminal trespassing, larceny, arson, and property damage; and 3) total crime, including all reported crimes [53]. Violent crime and property crime groupings were classified as such, based on the South Australian Department of Justice Australian National Classification of Offences [53]. These groupings include only those offences that were reported to the police or identified and recorded by police officers. Reported crimes do not necessarily result in a criminal conviction. Additionally, the spatial locations of reported crimes reflect where the offences were reported to have taken place; they do not reflect the residential location of those reported to have committed the offences nor those who reported the offences. Crime rates were ascribed to participants based on their SLA of residence, and standardised prior to analyses.

#### Potential mediator

Walking behaviour, expressed as time (in minutes) spent walking for sport, recreation or fitness over the previous week, was self-reported by participants using a single questionnaire item. This question has been used previously in Australia [54], and has substantial test-retest reliability (intraclass correlation = 0.78 (95% confidence interval 0.70–0.84)) [55]. Walking is a common form of physical activity among adults, and can contribute significantly to overall physical activity levels [56].

#### Covariates

Participant age, educational attainment (assessed as less than Bachelor’s degree or Bachelor’s degree or higher), and household income (assessed as AUD $20,000 or less, $20,001 to $60,000, or greater than $60,000) were entered as covariates in all models. Additionally, a measure of area-level socioeconomic status was included in all models to account for potential confounding by area socioeconomic status. Median weekly household income was extracted at the SLA level from the 2006 Australian Bureau of Statistics Census of Population and Housing [46], and ascribed to each participant based on the SLA in which they resided. Covariates were selected based on their potential to confound associations between crime and metabolic syndrome, and with consideration for issues of multicollinearity and over-adjustment.

### Statistical analysis

Four sets of analyses were conducted to test the research aims. All analyses were stratified by gender and were conducted using SAS (version 9.2; SAS Institute Inc., Cary, NC, USA). Statistical significance was set at α = 0.05.

First, descriptive statistics were used to describe the characteristics of the sample. Differences between men and women on these characteristics were assessed using chi-square tests, independent samples *t*-tests, and Wilcoxon-Mann-Whitney tests for non-normally distributed data.

Second, Pearson product-moment correlation coefficients were used to assess correspondence between area-level reported crime and individual-level perceived crime. The magnitude of correlation coefficients was classified according to Hemphill [57]: *r* < 0.2 small; *r* = 0.2 to 0.3 medium; *r* > 0.3 large.

Third, associations between crime measures and metabolic syndrome were first tested in separate models. Then, in light of the expected overlap between perceived and objective measures, their independent relationships with metabolic syndrome were evaluated by adding separately each of the objective measures to the perceived crime model. These models aimed to assess whether perceived or objective crime measures were more or less strongly related to metabolic syndrome, accounting for the influence of the other.

A final set of analyses was conducted to test the indirect effect (i.e., mediation) of crime on metabolic syndrome through walking behavior. The traditional approach to testing mediation was implemented in this study [58], where 1) the association between crime and walking, and then 2) the association between walking and metabolic syndrome accounting for crime, were estimated. Where both associations were statistically significant, indirect effects were tested using a nonparametric bootstrapping procedure [59] that estimates the sampling distribution of the indirect effect and the corresponding 95% confidence interval (CI) for a large number of samples (n = 20,000). This is a powerful method for assessing indirect effects, particularly where the indirect effect is not normally distributed and the outcome is binary [60]. Indirect effects were considered statistically significant when the 95% CI did not include zero [60]. All model sets included all potential confounders.

Generalised estimating equations (GEE) logistic regression was used in models where metabolic syndrome was the dependent variable. GEE Poisson regression was used to estimate the associations between crime and walking, where walking was the dependent variable. GEE logistic regression estimates were expressed as odds ratios (ORs), and GEE Poisson regression estimates were expressed as relative risks (RRs), both with corresponding 95% CIs. The GEE approach produces robust standard errors which account for the correlation of observations within clusters (i.e., participants within SLAs) [61].

## Results

### Sample description

A total of 1,530 participants had complete data for perceptions of neighbourhood crime and for defining the metabolic syndrome. Those with missing socio-demographic, walking, or residential location information (n = 125), residing outside the Adelaide metropolitan area at Wave 2 (n = 91), moved residence between the clinic visit and the telephone follow-up survey (n = 113), or resided in an SLA in Adelaide with less than three participants of the same gender (n = 29), were excluded. The final analytic sample included 540 men clustered within 24 SLAs (median *n* per SLA = 21, interquartile range = 24.8), and 632 women clustered within 25 SLAs (median *n* per SLA = 25, interquartile range = 31).

Table 1 presents the socio-demographic and area-level characteristics of the sample for men and women separately.

**Table 1:** Individual socio-demographic and area-level characteristics, by gender.

	Men (n = 540)		Women (n = 632)	
	Mean/Median or %	SD/ IQR	Mean/Median or %	SD/IQR
***Person-level characteristics***				
**Age** (years)^a^	56.2	14.2	54.3	13.8
**Education level**				
Less than Bachelor's degree	88.7%		85.1%	
Bachelor's degree or higher	11.3%		14.9%	
**Annual household income**				
Less than AUD$20,001	18.5%		26.1%	
AUD$20,001 to AUD$60,000	51.9%		46.2%	
More than AUD$60,000	29.6%		27.7%	
**Metabolic syndrome**	43.5%		29.6%	
**Perceived crime standardised factor score**^a^	-0.2	0.9	0.3	1.0
***Area-level characteristics***				
**Median weekly household income** (AUD)^a^	830.94	132.69	836.64	129.99
**Total crime rate** per 1,000 persons^b^	174.9	149.4	168.5	105.8
**Violent crime rate** per 1,000 persons^b^	12.8	11.7	12.3	8.9
**Property crime rate** per 1,000 persons^b^	97.8	73.4	103.6	61.1

SD: Standard Deviation. IQR: Interquartile Range.

^a^ Mean and SD reported.

^b^ Median and IQR reported.

### Correspondence between perceived and objective crime measures

For men, correspondence between perceived and objective crime measures was limited. Correlation coefficients between perceived crime and objective measures of total crime (r = 0.19; 95% CI = 0.11 to 0.27), violent crime (r = 0.22; 95% CI = 0.14 to 0.30) and property crime (r = 0.17; 95% CI = 0.08 to 0.25) were small to moderate. Levels of correspondence for all pairings of perceived and objective crime measures were greater for women than men. For women, correlation coefficients between perceived crime and objectively-measured total crime (r = 0.28; 95% CI = 0.21 to 0.35), violent crime (r = 0.33; 95% CI = 0.25 to 0.39) and property crime (r = 0.27; 95% CI = 0.19 to 0.34) were moderate to large. For both men and women, the correlation of greatest magnitude was that for the relationship between perceived crime and objectively-measured violent crime.

### Associations between crime and metabolic syndrome

Models testing each crime measure in relation to metabolic syndrome separately (Table 2) revealed that, among men, violent and total crime rates were associated with metabolic syndrome. Among women, only perceptions of neighbourhood crime were associated with metabolic syndrome.

**Table 2:** Associations between crime and metabolic syndrome, by gender.

	Men (n = 540)	Women (n = 632)
Independent variable	OR (95% CI)	OR (95% CI)
Perceived Crime	1.06 (0.89 to 1.27)	1.34 (1.14 to 1.59)
Total Crime	1.18 (1.06 to 1.30)	1.06 (0.87 to 1.29)
Violent Crime	1.21 (1.12 to 1.32)	1.05 (0.82 to 1.34)
Property Crime	1.14 (1.00 to 1.29)	1.01 (0.83 to 1.23)

OR: Odds Ratio. CI: Confidence Interval.

Note: All models adjusted for participant age, income, education, and area-level income.

Table 3 presents independent associations between perceived and reported crime variables and metabolic syndrome, where perceived crime was entered into models simultaneously with each reported crime variable.

**Table 3:** Independent associations between perceived and reported crime and metabolic syndrome, by gender.

**Men (n = 540)**			
**Independent Variable**	**Model a**	**Model b**	**Model c**
Perceived Crime	1.03 (0.85 to 1.25)	1.02 (0.83 to 1.24)	1.04 (0.86 to 1.26)
Total Crime	1.17 (1.04 to 1.32)		
Violent Crime		1.21 (1.08 to 1.35)	
Property Crime			1.13 (0.98 to 1.30)
**Women (n = 632)**			
**Independent Variable**	**Model a**	**Model b**	**Model c**
Perceived Crime	1.35 (1.14 to 1.60)	1.36 (1.15 to 1.61)	1.36 (1.14 to 1.62)
Total Crime	0.97 (0.79 to 1.18)		
Violent Crime		0.94 (0.77 to 1.14)	
Property Crime			0.92 (0.76 to 1.13)

Notes: Estimates reported are odds ratios with corresponding 95% confidence intervals. All models are adjusted for potential confounders, including participant age, income, education, and area-level income. Models combine perceived crime with either total (a), violent (b) and property crime (c).

With the inclusion of walking in Models a, b and c (Table 3), the associations between crime measures and metabolic syndrome were reduced by no more than 1% from estimates reported in Table 3 for both men and women (results not shown).

### Mediation by walking

Among men, no measure of crime was associated with walking, thus the indirect effect of crime on metabolic syndrome through walking were not tested for men. Among women, perceived neighbourhood crime and property crime rates were inversely associated with time spent walking (Table 4). Further, among women, walking was associated with metabolic syndrome, accounting for perceived and objective crime (OR 0.96, 95% CI 0.93–0.99), thus the criteria for testing indirect effects were met. There was an indirect effect of perceived crime on metabolic syndrome through walking (95% CI 0.0003–0.0073), and of property crime on metabolic syndrome through walking (95% CI 0.0001–0.0063).

**Table 4:** Associations between perceived and reported crime and walking, by gender.

	Men (n = 540)	Women (n = 632)
Independent variable	RR (95% CI)	RR (95% CI)
Perceived Crime^a^	0.88 (0.76 to 1.02)	0.86 (0.77 to 0.96)
Total Crime^b^	0.97 (0.81 to 1.17)	0.89 (0 .80 to 1.00)
Violent Crime^b^	0.92 (0.77 to 1.11)	0.94 (0.84 to 1.05)
Property Crime^b^	0.97 (0.80 to 1.16)	0.88 (0.79 to 0.98)

RR: Relative Risk. CI: Confidence Interval.

^a^ Models adjusted for participant age, income, education, area-level income, and total crime rates.

^b^ Models adjusted for participant age, income, education, area-level income, and perceived crime.

## Discussion

This study demonstrated gender-specific associations between objective neighbourhood crime rates, perceptions of neighbourhood crime, and clinically-measured metabolic syndrome in an urban Australian population. Objective crime rates were associated with metabolic syndrome among men, whereas among women, perceived crime was associated with metabolic syndrome. For both men and women, the correspondence between objective and perceived crime measures was generally small to moderate. Among women, this study found indirect effects of perceived crime on metabolic syndrome, and property crime on metabolic syndrome, through walking behaviour.

Low to moderate correspondence between area crime rates and perceptions of crime found in this study are consistent with that for area-level objective crime and individual-level perceived crime as previously reported (r = 0.20 to 0.25) [62]. Three additional previous studies [8, 10, 63] have reported a similarly low level of agreement between objective crime data and perceived measures of crime or safety, with measures expressed at the individual level. The low to moderate agreement found in this study between perceived and objective measures may be a result of the different spatial scales of the measures. In this study, perceived crime was assessed for participants’ “local area”, with “local” being defined as within a 10–15 minute walk from home (approximately 5 km^2^), whereas objective crime measures were expressed at a much larger scale, on average 33.2 km^2^ [52].

Eliciting resident perceptions of neighbourhood safety has been proposed as a "relatively simple method for identifying at-risk environments and persons at particularly high risk for obesity in those settings" [17]. The differential relationships between objective and perceived crime and metabolic syndrome observed here would suggest that perceptions of neighbourhood safety may not appropriately identify an at-risk environment for men. In this study, only actual rates of violent crime and total crime were related to metabolic syndrome in men. In women, only perceived crime was associated with metabolic syndrome. The novel, gender-specific finding in this study of differential relationships between perceived and objective measures of crime and metabolic syndrome is important. The associations reported here were robust to adjustment for individual- and area-level socioeconomic status, and to inclusion of both perceived and objective crime measures in the same statistical models.

Previous research concerned with relationships between crime and cardiometabolic outcomes [14–18, 21, 64] has tended to rely on either perceived or objective measures of crime, but not both. With one exception [21], studies using perceptual measures of crime have tended to examine such measures in relation to self-reported health outcomes [16–18, 64], introducing single-source bias. Moreover, just one of these studies [15] examined gender-specific relationships between crime and a measured cardiovascular outcome, finding similarly positive associations between neighbourhood crime rates and coronary heart disease hospitalisation in both men and women. This particular study, however, did not account for potential confounding by area socioeconomic status, nor did it include a measure of perceived crime.

The importance of including both perceived and objective neighbourhood measures in place-health research is highlighted by two key results in the present study. First is the demonstration of a limited correspondence between perceived and objective crime measures, this being consistent with previous research [8, 10, 62, 63]. Second is the identification of unique and independent gender-specific effects of both objective and perceived measures of crime in relation to a clinically-measured cardiometabolic outcome, metabolic syndrome.

The patterns of association found between crime and metabolic syndrome in this study lend support to the notion of gender-specific mechanisms by which social phenomena such as local-area crime and perceptions of neighbourhood crime relate to health, in this case, cardiometabolic risk. The results also help explain gender differences in crime-health associations. In this study, it was hypothesised that chronic stress may be a mechanism by which neighbourhood crime is related to cardiometabolic outcomes among men, and that a cognitive-behavioural pathway might act to link perceptions of crime to cardiometabolic outcomes among women. Testing mediation through the analysis of direct and indirect effects can be motivated by a desire to strengthen evidence that any main effects found are potentially causal, and to test pathway-specific hypotheses [43]. In this study with a reasonable if limited sample size, there was an indirect effect of perceived crime on metabolic syndrome, and of property crime on metabolic syndrome, via walking behaviour among women. Walking appears not to mediate crime-metabolic syndrome associations for men. Walking in this study was self-reported, and while time spent walking has demonstrated acceptable correlations with accelerometer or pedometer measurements [65], objectively-assessed physical activity may have improved the rigour of these analyses. However, only self-reported measures were available for this research. It may also be that walking for recreation, sport or leisure is less likely to mediate crime-health associations for men compared to walking for transport. Other neighbourhood-based physical activity could potentially be even more important than walking, for example, through use of local recreational and sporting facilities. While perceived safety has been associated with lower levels of leisure-time walking among women [66], recent evidence suggests that walking for transport, but not walking for leisure, may be related to objective crime rates [67]. The partial mediating effect of walking in crime-metabolic syndrome associations among women, and no such effect among men, indicates the potential for the contribution of other mechanisms linking crime to metabolic syndrome. For instance, it is possible that a pathway implicating chronic stress operates to link local-area crime rates to cardiometabolic health [29]. Unfortunately, alternate potential mediators could not be tested in this study due to a lack of data. Additionally, it is possible that walking limitations among some participants contributed to the results found in the present study. As it was hypothesised that factors other than walking may contribute to mediating crime-health associations, participants with physical limitations were not excluded from this research. Future research might consider excluding persons with physical limitations to investigate this in more detail. Further research in differing social and spatial contexts, with longitudinal data, and with attention to other potential mediating factors, will contribute to understanding these gender differences in relationships between crime and cardiometabolic risk.

The examination of both objective and perceived measures of crime in relation to a clinically measured cardiometabolic outcome in a representative population-based sample is a strength of this study. Several limitations, however, may bear on the results observed. Firstly, the cross-sectional nature of the study is a significant shortcoming. In order to interpret the findings particularly with regard to the mediation analysis within a causal framework, ideally a causal mediation approach would be used in the analysis, such as described by Valeri and VanderWeele [68] among others. Unfortunately, the data available for this study could not meet the required assumptions for a causal mediation approach with regard to unmeasured confounding and temporality of measures; thus, the results of this study regarding indirect effect of crime on metabolic syndrome via walking behaviour must be interpreted with caution. It is possible that metabolic syndrome precedes reduced walking behavior, as several studies have shown that adiposity is causally related to decreased physical activity in longitudinal studies of children [69–71]. It is also possible that the results presented are biased by residual confounding or unmeasured confounding, a common issue in observational studies [72]. Secondly, a key consideration in place and health research is the spatial unit employed, for the use of different scales for expressing crime and disorder could yield different relationships [29]. Ideally, crime-health relationships would have been evaluated for multiple, particularly smaller, spatial units. Sensitivities around the release of South Australian crime data precluded the possibility of analyses at a finer resolution than the SLA. SLAs across metropolitan Adelaide are also highly variable in area (3.9 to 339 km^2^). If the positioning of such administratively-defined boundaries were modified, different associations between area-level crime and health outcomes may arise. These issues, relating to the modifiable areal unit problem [73], limit the direct comparability of actual crime with the measure of perceived crime in the local area. Thirdly, it is possible that participants with better cardiometabolic and behavioural risk profiles may have self-selected into more healthful, safer neighbourhoods. Factors that may lead to biases regarding participant selective settlement into more healthful neighbourhoods, such as neighbourhood preferences, were not able to be accounted for in this research. Fourthly, the measure of walking used in this study was self-reported, the only type of measure available for this research, and may not accurately reflect actual levels of walking or physical activity (time or intensity) compared to objective measures such as accelerometry [65]. Last, it is widely acknowledged that reported offences data underestimate actual levels of crime, as not all crimes are reported [74]. It is also the case that reporting of crime is influenced by public policy, whereby strategies to encourage reporting of certain types of criminal activity such as rape, or increased police attention to specific crimes, for instance, driving offences, can result in higher rates of reported crime [74]. Despite these limitations, reported crime rates are known to have a similar geographic heterogeneity to victimisation rates [75] and are no more susceptible to biases in reporting and classification than are objective crime data obtained from police arrests and victimisation surveys [76].

## Conclusions

The present study provides novel evidence that objective neighbourhood crime rates among men, and perceptions of neighbourhood crime among women, are related to clinically-measured cardiometabolic risk, expressed as the metabolic syndrome. This study further contributes new empirical knowledge regarding walking behaviour as one potential mechanism linking neighbourhood crime to cardiometabolic risk among adult women, although these results are interpreted with caution given the cross-sectional nature of the study. The results confirm the importance of including objective and perceived measures of environmental exposures, in this case crime, when examining relations with health outcomes. It also highlights the importance of considering potential gender differences in assessing the social-health phenomena.

The findings generated by this study have relevance for policy makers charged with developing strategies to reduce levels of crime and to improve perceptions of crime and safety as part of ecological initiatives to reduce cardiometabolic risk (and related lifestyle diseases) at the population level. For instance, government-led interventions aimed at increasing the educational, recreational, and occupational opportunities available at the local level may be important in facilitating healthy community life, potentially contributing to lower rates of crime. Social policy focused on community re-development to encourage participation in community life, enabling the creation and maintenance of local social networks and increased collective efficacy, may further lead to reduced crime and more positive perceptions of crime and safety. The gender differences identified in this study are relevant to the service providers responsible for designing and delivering gender appropriate public health and policy interventions [77]. For instance, improving perceptions of crime for women may require a focus on relational strategies such as increasing social connectedness through community walking groups, whereas for men the greatest impact may be achieved through the lowering of rates of objective crime. In combination, tailored policies and interventions may have wide-reaching effects on population health and well-being.
